# Establishment and validation of a novel risk model for estimating time to first treatment in 120 patients with chronic myelomonocytic leukaemia

**DOI:** 10.1007/s00508-018-1315-2

**Published:** 2018-01-30

**Authors:** Florian Huemer, Lukas Weiss, Viktoria Faber, Daniel Neureiter, Alexander Egle, Klaus Geissler, Daniela Voskova, Armin Zebisch, Sonja Burgstaller, Angelika Pichler, Reinhard Stauder, Wolfgang Sperr, Alois Lang, Michael Pfeilstöcker, Sigrid Machherndl-Spandl, Margarete Stampfl, Richard Greil, Lisa Pleyer

**Affiliations:** 1 Department of Internal Medicine III with Hematology, Medical Oncology, Hemostaseology, Infectious Disease, Rheumatology, Oncologic Center, Laboratory of Immunological and Molecular Cancer Research, Paracelsus Medical University Salzburg, Muellner Hauptstraße 48, 5020 Salzburg, Austria; 2 Cancer Cluster Salzburg, Salzburg, Austria; 3 Institute of Pathology, Paracelsus Medical University, Salzburg, Austria; 4 5th Department of Medicine, Hospital Hietzing, Vienna, Austria; 5 Clinical Division of Internal Medicine 3‑Hematology and Oncology, Kepler University Hospital, Linz, Austria; 6 Division of Haematology, Department of Internal Medicine, Medical University of Graz, Graz, Austria; 7 Department of Internal Medicine IV, Klinikum Wels-Grieskirchen, Wels, Austria; 8 Department for Haematology and Oncology, LKH Hochsteiermark, Leoben, Austria; 9 Department of Internal Medicine V, Innsbruck Medical University, Innsbruck, Austria; 10 Department of Internal Medicine I, Division of Haematology and Haemostaseology, Medical University of Vienna, Vienna, Austria; 11 Department of Internal Medicine, Landeskrankenhaus Feldkirch, Feldkirch, Austria; 12 3rd Medical Department for Haematology and Oncology, Hanusch Hospital, Vienna, Austria; 13 Department of Haematology and Oncology, Elisabethinen Hospital, Linz, Austria; 14 Department of Internal Medicine 2, Donauspital—SMZO, Vienna, Austria

**Keywords:** Azacitidine, CMML, Hydroxyurea, Prognostic factors, Austrian Registry on Hypomethylating Agents

## Abstract

Chronic myelomonocytic leukaemia is a rare disease and data on the treatment are often extrapolated from myelodysplastic syndrome studies. Although several scores exist for the prognosis of overall survival in chronic myelomonocytic leukaemia, so far there is no designated score for the prediction of the time to first treatment. We tested clinical parameters and cytogenetic information for their ability to predict the time to first treatment in our single center cohort of 55 unselected consecutive chronic myelomonocytic leukaemia patients. In multivariate analysis we identified elevated lactate dehydrogenase (≥223 U/l), higher bone marrow blast percentage (≥7.5%) and thrombocytopenia (<55 G/l) at initial diagnosis as the most relevant parameters for the time to first treatment. Using these three parameters we developed a risk score that efficiently estimates the time to treatment initiation with azacitidine or hydroxyurea (*p* < 0.001; log-rank). In the high-risk group (≥2 risk factors) 85% of patients required treatment within 1 year, whereas this was the case in 48% in the intermediate-risk (1 risk factor) and in 0% in the low-risk group (0 risk factors). Our risk model was validated in an external test cohort of 65 patients and may serve as a simplified and easily applicable tool for identifying patients who may not require early treatment initiation.

## Introduction

Chronic myelomonocytic leukaemia (CMML) is a clonal hematopoietic stem cell disorder that is regarded as myeloproliferative/myelodysplastic overlap disorder according to the 2016 revision to the World Health Organization (WHO) classification of myeloid neoplasms and acute leukaemia. Besides the absence of the Philadelphia chromosome and/or the BCR-ABL fusion gene, evidence of dysplasia in at least one myeloid lineage and less than 20% myeloblasts, monoblasts or promonocytes in the peripheral blood or bone marrow and a persistent monocytosis >1000/µl with monocytes accounting for ≥10% of the white blood cell count and exclusion of secondary causes thereof are required to establish the diagnosis of CMML. If myelodysplastic features are missing, the detection of an acquired clonal or molecular genetic abnormality in hematopoietic stem cells and/or a persistent monocytosis for more than 3 months without other causes can still lead to the diagnosis of CMML. Rearrangements of the platelet-derived growth factor receptor alpha (PDGFRA) gene, of the platelet-derived growth factor receptor beta (PDGFRB) gene and the fibroblast growth factor receptor 1 (FGR1) gene or PCM1-JAK2 fusions must be excluded if eosinophilia is present [1].

While cytogenetic abnormalities are only found in approximately 30% of patients with CMML, molecular abnormalities have been reported in up to 90% of CMML cases [2–5]. Prognostic tools, such as the MD Anderson prognostic score (MDAPS) divide treatment-naïve CMML patients into the risk groups “low”, “intermediate-1”, “intermediate-2” and “high” with a median overall survival (OS) of 24, 15, 8 and 5 months, respectively [6]. Several other CMML-specific scores, e.g. modified MDAPS (MDAPS M1), CMML-specific prognostic scoring system (CPSS) and the Mayo prognostic model, are used to predict OS [7–9]. The Düsseldorf score, international prognostic scoring system (IPSS) and the revised IPSS (IPSS-R) were primarily applied to estimate OS in MDS but also included myelodysplastic CMML (MD-CMML) patients [10–12]. Molecular abnormalities, such as ASXL1, NRAS, RUNX1 and SETBP1 mutations impact on OS and have already been included in molecular prognostic risk models in CMML [5, 13, 14].

Current therapeutic options in CMML are limited. A best supportive care strategy may include transfusion of blood products, administration of erythropoiesis-stimulating agents or myeloid growth factors, antibiotics, antiviral medication, iron chelation therapy as well as cytoreduction with either hydroxyurea or etoposide [15]. In the only randomized clinical trial performed and published in CMML to date including 105 patients, oral hydroxyurea proved to be superior to oral etoposide in terms of response rate and median OS [16]. In retrospective analyses, the hypomethylating agents azacitidine and decitabine yielded a median OS of 13.2 and 19.0 months in CMML, respectively [17, 18]. The approval of both substances by the U.S. Food and Drug Administration (FDA) for the treatment of CMML is based on the results of two phase III trials, albeit the number of included CMML patients (14 in each study) was low [19, 20]. In contrast to the U.S. the European Medicines Agency (EMA) only approved azacitidine for a subset of CMML patients, for those with MD-CMML and a bone marrow blast percentage of 10–29%, which therefore includes a subset of patients who fulfil the criteria of acute myeloid leukaemia according to the WHO classification, namely those with 20–29% bone marrow blasts. Up to now, decitabine has neither been approved for the treatment of CMML, nor for the treatment of myelodysplastic syndromes (MDS) in Europe. Even with intensive chemotherapy protocols the median OS does not exceed 44 weeks and allogeneic stem cell transplantation represents the only curative treatment approach in CMML [21, 22].

Treatment criteria in CMML have not been established so far and therefore treatment indications are mainly based on expert opinion or consensus statements from expert panels including severe anemia (hemoglobin <10 g/dl), blast percentage >5% in the peripheral blood, immature myeloid cells including myeloblasts, promyelocytes, myelocytes and metamyelocytes >10% in the peripheral blood, platelet count <50 G/l, white blood cell count >30 G/l, extramedullary disease manifestations, presence of B‑symptoms and symptomatic splenomegaly [15, 23]. According to the current National Comprehensive Cancer Network guidelines for MDS, which subsume CMML, symptomatic anemia, clinically relevant thrombocytopenia and/or neutropenia or increased bone marrow blasts demonstrate potential treatment indications for CMML-specific therapy [24].

Apart from the abovementioned treatment indications and due to the heterogeneity of this disease as well as the broad spectrum of the clinical course of CMML, the time to first treatment (TTFT) with intensive chemotherapy regimens, hypomethylating agents or cytoreductive therapy, such as hydroxyurea varies considerably among patients. Defining treatment indications as well as choosing the optimal time point for treatment initiation pose common challenges in clinical practice, and analyses of a potential effect of differences in TTFT on OS are scarce or lacking in CMML.

The aim of this single centre retrospective study was (a) to evaluate which parameters at the initial diagnosis of CMML were relevant to the time point of treatment initiation based on established treatment indications in clinical practice at the center in Salzburg, (b) to propose a simplified risk model for TTFT in CMML, and (c) to validate this risk model in an external test cohort.

## Patients and methods

This retrospective analysis was approved by the Ethics Committee of the provincial government of Salzburg, Austria (reference number 415-EP/39/11) and was based on the data of 55 unselected consecutive CMML patients (training set) diagnosed and/or treated at our tertiary oncology center in Salzburg, Austria, between 2004 and 2015. Those CMML patients who received azacitidine during the course of the disease were included in the Austrian Registry on Hypomethylating Agents (NCT01595295) of the working group on pharmaceutical tumor treatment (Arbeitsgemeinschaft Medikamentöse Tumortherapie, AGMT; www.agmt.at). The external independent validation set consisted of 65 CMML patients from 12 Austrian hospitals included in the Austrian Registry on Hypomethylating Agents, with the majority (*n* = 60) derived from the data base of the Austrian Registry on Hypomethylating Agents. All patients alive at the time point of data acquisition signed an informed consent to allow the collection of personal data. The diagnosis was established according to the 2008 WHO classification of tumors of hematopoietic and lymphoid tissues [25]. The OS was calculated from the date of first diagnosis until date of death or date of last known follow-up, TTFT was defined as the time period between initial diagnosis and first CMML-specific treatment with either hydroxyurea or azacitidine.

### Statistics

Estimates on TTFT distributions were based on the Kaplan-Meier method and the log-rank test was used to compare Kaplan-Meier survival curves. We analyzed the impact of various clinical baseline factors and cytogenetic abnormalities at initial diagnosis on TTFT, which in part had already been incorporated into established prognostic scores for OS [6–12]. The optimal cut-offs for discerning the treatment status (untreated or treated) of patients at the end of the follow-up time were calculated based on receiver operating characteristics (ROC) analyses and the Youden index J, which represents the maximum of sensitivity_c_+specitivity_c-1_ for all cut points in the ROC curve [26]. In order to avoid variable redundancy, the WHO classification was not included in the univariate and multivariate analysis, as this is determined by the percentage of bone marrow blasts and peripheral blood blasts: CMML-1 (blasts <5% in the peripheral blood and blasts <10% in the bone marrow) and CMML-2 (peripheral blood blasts 5–19% and/or 10–19% bone marrow blasts[25]). Parameters which proved statistically significant in univariate analysis (*p* < 0.05) were included in multivariate analysis. Statistical analyses were carried out using the IBM® SPSS® statistics software, version 20.

## Results

### Baseline characteristics

The baseline characteristics of 55 unselected consecutive CMML patients diagnosed and/or treated at the center in Salzburg (training set) and of 65 patients included in the validation set are depicted in Table 1.

**Table 1:** Baseline characteristics of CMML patients in the training set and the validation set

Baseline characteristic	Training set *N* = 55 (%)	Validation set *N* = 65 (%)	*p*-value^a^
*Sex*			
Male	32 (58)	41 (63)	0.584
Female	23 (42)	24 (37)	
*Median age at diagnosis (years)*	75	71	0.003
(Range)	(38–96)	(55–84)	
*WHO subtype*			
CMML-1	41 (75)	49 (75)	0.916
CMML-2	14 (25)	16 (25)	
*FAB subtype*			
MD-CMML	32 (58)	33 (51)	0.417
MP-CMML	23 (42)	32 (49)	
*Cytogenetics* ^*b*^			
Normal karyotype	33 (80)	34 (60)	0.029
Abnormal karyotype	8 (20)	23 (40)	
*Red blood cell transfusion-dependence*			
No	44 (80)	51 (78)	0.836
Yes	11 (20)	14 (22)	
Pretreatment with ESA			
No	41 (75)	52 (80)	0.476
Yes	14 (25)	13 (20)	
*First-line treatment substance* ^*b*^			
Azacitidine	21 (62)	47 (80)	0.061
Hydroxyurea	13 (38)	12 (20)	

*FAB* French American British classification, *ESA* erythropoiesis-stimulating agents, *WHO* World Health Organization, *MD-CMML* myelodysplastic chronic myelomonocytic leukaemia, *MP-CMML* myeloproliferative chronic myelomonocytic leukaemia

^a^2-sided χ^2^-test (Pearson)

^b^Percentage of classifiable patients

#### Training set

The median age at diagnosis was 75 years (range 38–96 years) with a male predominance (58%). According to the FAB classification 58% had MD-CMML and 75% and 25% were categorized as CMML-1 and CMML-2, respectively. The karyotype analysis was available in 75% at the initial diagnosis, and an abnormal karyotype was detectable in 20% of evaluable patients. According to the MDAPS, which is specific for CMML, 26% of our patients were classified as higher-risk (22% intermediate-2, 4% high risk). At the time of data analysis, 38% and 24% of patients had received azacitidine and hydroxyurea as first-line treatment, respectively, whereas 38% were treatment-naïve. The indications for initiation of first-line treatment in each individual patient are shown in Table 2. Of note, only 47% of patients with MD-CMML received front-line treatment with either azacitidine (87%) or hydroxyurea (13%), and one patient received azacitidine as a subsequent therapy after hydroxyurea, whereas 83% of patients with myeloproliferative CMML (MP-CMML) received treatment with either azacitidine (42%) or hydroxyurea (58%). Among those MP-CMML patients who had initially been treated with hydroxyurea, 55% were subsequently switched to azacitidine. A female patient (38 years) with the myeloproliferative CMML variant received an unrelated matched donor allogeneic stem cell transplantation after bridging therapy with hydoxyurea followed by azacitidine and transformation to secondary acute myeloid leukaemia. Only 1 out of 21 treatment-naïve patients presented with a hemoglobin level below 8 g/dl at initial diagnosis and formally was a candidate for azacitidine initiation. Due to advanced age and an excellent response to erythropoiesis-stimulating agents, hypomethylating therapy was not initiated. Out of 14 treatment-naïve patients 6 had an initial hemoglobin level in the range of 8–10 and of 10–14 g/dl, respectively, and were not considered red blood cell transfusion-dependent. Among patients who presented with anemia or developed anemia during the course of the disease, 14 patients (26%) initially received erythropoiesis-stimulating agents (Table 2). A total of 40 (73%) patients had died at the time point of data analysis (11 March 2016). The median TTFT was 10.7 months (95% confidence interval CI 0.0–25.6 months), the median follow up was 44.3 months (95% CI 36.3–52.3 months), median OS was 28.4 months (95% CI 19.7–37.1). Among evaluable patients 10 (20%) developed a transformation into acute leukaemia, determined by bone marrow aspiration and/or biopsy and/or peripheral blood blast count ≥20%.

**Table 2:** Patient characteristics of the training set (*n* = 55) at treatment start with azacitidine, hydroxyurea and/or erythropoiesis-stimulating agents

Patient	First-line treatment	Salzburg Risk Model	ESA	Anemia (<10 g/dl)	Leukocytosis (>20 G/l)	Thrombocytopenia (<50 G/l)	BM blasts (≥10%)	Peripheral blood blasts (≥5%)
1	AZA	Intermediate	–	X	X	–	–	–
2	AZA	High	–	–	–	X	X	–
3	AZA	High	–	X	X	X	–	–
4	AZA	High	–	X	–	–	X	–
5	AZA	High	–	X	X	–	–	–
6	AZA	High	–	X	–	X	–	–
7	AZA	Intermediate	–	X	–	–	–	–
8	AZA	Intermediate	–	–	–	X	–	–
9	AZA	High	–	X	–	X	X	–
10	AZA	High	–	X	–	X	X	X
11	AZA	High	–	–	X	X	X	–
12	AZA	Intermediate	–	X	X	–	–	–
13	AZA	Intermediate	–	–	X	X	–	X
14	AZA	Intermediate	–	X	–	X	–	–
15	AZA	High	–	X	X	–	X	X
16	AZA	Intermediate	X	X	–	–	–	–
17	AZA	High	–	–	–	X	–	–
18	AZA	Intermediate	–	–	X	–	–	–
19	AZA	High	–	X	X	–	X	X
20	AZA	High	–	–	–	X	–	–
21	AZA	High	X	–	–	X	–	X
22	HU	High	–	–	X	X	X	–
23	HU	Intermediate	–	X	X	–	–	X
24	HU	Intermediate	–	–	X	–	–	–
25	HU	Not available	–	–	X	–	X	–
26	HU	High	–	–	X	X	–	–
27	HU	High	–	X	X	–	–	X
28	HU	Intermediate	X	–	X	–	–	–
29	HU	Intermediate	–	X	X	X	–	–
30	HU	High	–	X	X	X	–	–
31	HU	Intermediate	–	X	X	X	–	–
32	HU	High	X	X	X	X	–	–
33	HU	Intermediate	X	X	X	–	–	–
34	HU	High	–	–	X	–	–	–
35	Naive	High	–	–	–	–	–	–
36	Naive	Intermediate	X	X	–	–	–	–
37	Naive	Low	X	X	–	–	–	–
38	Naive	Low	X	X	–	–	–	–
39	Naive	Low	–	–	–	–	–	–
40	Naive	Intermediate	–	–	–	–	–	–
41	Naive	Intermediate	–	–	–	–	–	–
42	Naive	Intermediate	X	X	–	–	–	–
43	Naive	Not available	–	–	–	–	–	–
44	Naive	Intermediate	X	X	–	–	–	–
45	Naive	Intermediate	–	–	–	–	–	–
46	Naive	Low	X	X	–	–	–	–
47	Naive	Low	X	X	–	–	–	–
48	Naive	Intermediate	–	–	–	–	–	–
49	Naive	Low	–	–	–	–	–	–
50	Naive	Low	–	–	–	–	–	–
51	Naive	Intermediate	–	–	–	–	–	–
52	Naive	Intermediate	X	X	–	–	–	–
53	Naive	Low	–	–	–	–	–	–
54	Naive	Intermediate	X	X	–	–	–	–
55	Naive	Low	–	–	–	–	–	–

*AZA* azacitidine, *HU* hydroxyurea, *ESA* erythropoiesis-stimulating agents, *BM* bone marrow, *X* indicates fulfilled treatment indication

#### Validation set

A total of 65 CMML patients were included in the external validation set. The median age was 71 years (range 55–84 years) with a male predominance (63%). The myelodysplastic variant was documented in 51% of patients and 75% were classified as CMML-1. Karyotype analysis at the initial diagnosis was available in 88% and abnormal karyotype was detectable in 40% of evaluable patients. For additional details see Table 1. At the time of data analysis (1 March 2017) 73% and 18% of patients had received azacitidine and hydroxyurea as first-line treatment, respectively, whereas the remaining 9% were treatment-naïve. A total of 49 (75%) of all patients had died at the time point of data analysis. The median TTFT was 3.0 months (95% CI 0.0–10.0 months), the median follow-up was 77.7 months (95% CI 21.7–133.7 months), median OS was 26.1 months (95% CI 18.3–33.9 months).

### Univariate and multivariate analysis of baseline factors for time to first treatment

First, we aimed at evaluating which parameters at the time point of the initial CMML diagnosis influence the TTFT in clinical practice. After the calculation of cut-off points for parameters measured on a continuous scale with ROC analyses and the Youden index, the following variables were associated with shorter TTFT in univariate analysis: the presence of immature myeloid cells in peripheral blood, white blood cell count (≥14.5 G/l), platelets (<55 G/l), absolute neutrophil count (≥6 G/l), absolute lymphocyte count (≥2.3 G/l), absolute monocyte count (≥2.8 G/l), lactate dehydrogenase (≥223 U/l), presence of peripheral blood blasts, bone marrow blast percentage (≥7.5%), red blood cell transfusion-dependence and the presence of B‑symptoms at initial diagnosis (Table 3). With the exception of sex, C‑reactive protein, palpable spleen and/or symptomatic splenomegaly and the presence of B‑symptoms, the tested parameters were included in the established prognostic scores [6–12]. In multivariate analysis, the following factors remained independently associated with TTFT: lactate dehydrogenase (≥223 U/l, relative risk [RR] 5.428, 95% CI 1.550–19.010, *p* = 0.008), bone marrow blasts (≥7.5%, RR 4.570, 95% CI 1.794–11.641, *p* = 0.001) and platelets (<55 G/l, RR 2.660, 95% CI 1.119–6.325, *p* = 0.027) (Table 4). Among the 55 CMML patients in the training set, 2 patients were not included in the multivariate analysis for TTFT because of missing data.

**Table 3:** Univariate analysis for time to first treatment, training set

Univariate analysis-Training set				
Parameter	*N*	RR	95% CI	*P*-value
*Sex*				
Female	23	1.596	0.811–3.142	0.176
Male	32			
*Cytogenetics*				
Normal	33	1.442	0.570–3.644	0.439
Abnormal	8			
*Immature myeloid cells*				
Yes	33	2.447	1.133–5.286	0.023*
No	20			
Hemoglobin	54	1.726	0.866–3.438	0.121
<11.6 g/dl	29			
≥11.6 g/dl	25			
*WBC*				
≥14.5 G/l	19	5.843	2.801–12.189	<0.001*
<14.5 G/l	35			
*Platelet count*				
<55 G/l	16	2.505	1.242–5.053	0.010*
≥55 G/l	38			
*Neutrophil count*				
≥6 G/l	26	2.779	1.380–5.595	0.004*
<6 G/l	28			
*Lymphocyte count*				
≥2.3 G/l	23	2.253	1.138–4.460	0.020*
<2.3 G/l	31			
*Monocyte count*				
≥2.8 G/l	25	4.427	2.102–9.321	<0.001*
<2.8 G/l	29			
Lactate dehydrogenase				
≥223 U/l	39	5.465	1.912–15.622	0.002*
<223 U/l	16			
CRP				
≥1 mg/dl	20	1.584	0.740–3.393	0.236
<1 mg/dl	22			
Peripheral blood blasts				
Yes	13	4.447	2.063–9.586	<0.001*
No	40			
*Bone marrow blasts*				
≥7.5%	16	2.621	1.305–5.264	0.007*
<7.5%	37			
*RBC transfusion dependence*				
Yes	11	2.183	1.014–4.699	0.046*
No	44			
*Palpable spleen and/or symptomatic splenomegaly*				
Yes	3	1.909	0.582–6.265	0.286
No	52			
*B-symptoms*				
Yes	6	2.707	1.098–6.677	0.031*
No	49			

*RR* relative risk, *CI* confidence interval, *RBC* red blood cell, CRP ,WBC

* *p*-value < 0.05

**Table 4:** Multivariate analysis for time to first treatment, training set

Multivariate analysis-Training set				
Parameter	*N*	RR	95% CI	*P*-value
*Immature myeloid cells*				
Yes	33	1.124	0.371–3.402	0.837
No	20			
*WBC*				
≥14.5 G/l	19	2.354	0.592–9.363	0.224
<14.5 G/l	34			
*Platelet count*				
<55 G/l	16	2.660	1.119–6.325	0.027*
≥55 G/l	37			
*Neutrophil count*				
≥6 G/l	26	1.425	0.380–5.344	0.599
<6 G/l	27			
*Lymphocyte count*				
≥2.3 G/l	23	1.475	0.506–4.298	0.476
<2.3 G/l	30			
*Monocyte count*				
≥2.8 G/l	25	1.289	0.389–4.271	0.678
<2.8 G/l	28			
*Lactate dehydrogenase*				
≥223 U/l	37	5.428	1.550–19.010	0.008*
<223 U/l	16			
*Peripheral blood blasts*				
Yes	13	2.576	0.989–6.707	0.399
No	40			
*Bone marrow blasts*				
≥7.5%	16	4.570	1.794–11.641	0.001*
<7.5%	37			
*RBC transfusion dependence*				
Yes	10	1.929	0.733–5.076	0.183
No	43			
*B-symptoms*				
Yes	6	1.120	0.409–3.065	0.826
No	47			

*RR* relative risk, *CI* confidence interval, *RBC* red blood cell, *WBC* white blood cell

*: *p*-value < 0.05

### Proposing a novel risk score for the prediction of time to first treatment

**Fig. 1:**
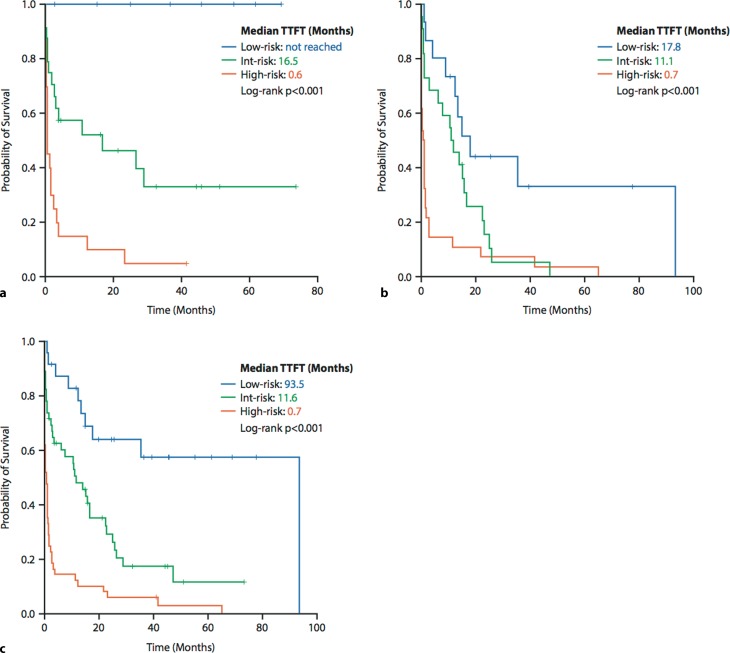
Time to first treatment (TTFT) according to the Salzburg Risk Model in the training set, validation set and full patient series. **a** TTFT in the training set (*n* = 53). The tick marks on the curves represent censored patients. **b** TTFT in the validation set (*n* = 65). **c** TTFT in the full patient series (training set + validation set, *n* = 118)

The three clinical parameters identified as being independently associated with TTFT in multivariate analysis were included into a TTFT risk model. One point was allocated for each risk factor at initial diagnosis: lactate dehydrogenase ≥223 U/l, bone marrow blasts ≥7.5%, and platelets <55 G/l. According to the sum of these points CMML patients were stratified into three subgroups: low risk (score = 0), intermediate risk (score = 1) and high risk (score ≥ 2). This score efficiently separated patients with differing risk profiles for TTFT (*p* < 0.001; log-rank): in the high-risk group 85% of patients required treatment within 1 year, whereas this was the case in 48% in the intermediate-risk and in 0% in the low-risk groups (Fig. 1a). The treatment indications as well as the risk group according to our TTFT risk model for each individual patient are shown in Table 2. First-line therapy with azacitidine was initiated due to anemia (<10 g/dl), thrombocytopenia (<50 G/l), leukocytosis (>20 G/l), bone marrow blast percentage (≥10) and/or peripheral blast percentage (≥5) in 62%, 57%, 43%, 33% and 24%, respectively. An increase in leukocytes (>20 G/l) was a prerequisite for front-line treatment with hydroxyurea in our CMML patients. Concomitantly, 54%, 46%, 15% and 15% of patients presented with anemia (<10 g/dl), thrombocytopenia (<50 G/l), bone marrow blast percentage (≥10) and/or peripheral blast percentage (≥5) at the time point of treatment initiation, respectively. Erythropoiesis-stimulating agents were applied in 44%, 33% and 10% of low-risk, intermediate-risk and high-risk patients according to our TTFT risk classification.

### Validation of novel risk score

Subsequently, our risk model was tested in an independent cohort of 65 CMML patients. The results were replicated and our model adequately discriminated TTFT among three risk groups on the basis of the three clinical parameters. In the high-risk group (≥2 risk factors) 89% of patients required treatment within 1 year, whereas this was the case in 54% in the intermediate-risk (1 risk factor) and in 27% in the low-risk groups (0 risk factors; *p* < 0.001; log-rank; Fig. 1b). We then applied the risk model to the full patient series including the training set (*n* = 53) and the validation set (*n* = 65) with a total of 118 patients: 87%, 52%, and 17% of patients in the high-risk group, intermediate-risk group and low risk group required treatment initiation with either azacitidine or hydroxyurea within the first year after initial diagnosis of CMML (*p* < 0.001; log-rank; Fig. 1c).

## Discussion

The optimal time point to initiate treatment in CMML may pose a challenge to the treating physician. For example, while there is a general consensus that symptomatic cytopenia in CMML demonstrates a treatment indication, initial management of the latter often involves application of erythropoiesis-stimulating agents or blood products without immediately initiating treatment with hypomethylating agents or chemotherapy. Whether such a treatment delay is associated with a better or worse clinical outcome has not been prospectively tested in CMML so far. In clinical practice at our center in Salzburg, the decision to start treatment with either hydroxyurea or azacitidine is mainly based on (i) expert panel recommendations, (ii) the National Comprehensive Cancer Network guidelines for MDS and (iii) the clinical presentation of the individual patients [15, 23, 24]. With an analysis of 55 unselected consecutive CMML patients we evaluated which baseline factors impact on treatment initiation with either azacitidine or hydroxyurea in CMML. By testing the influence of individual parameters applied in established prognostic scores and further variables, lactate dehydrogenase, bone marrow blast percentage and platelets at the initial diagnosis were significantly associated with TTFT. Because a single cut-off was desirable for further statistical analyses and comparison with other dichotomous variables, we calculated cut-offs for parameters measured on a continuous scale with ROC analysis and the Youden index. These cut-off values were in line with a clinically relevant graduation, for example, the calculated cut-off for lactate dehydrogenase (≥223 U/l) represents the upper limit of normal and the cut-off for the platelets (<55 G/l) closely matches the definition of grade 3 thrombocytopenia according to the Common Terminology Criteria for Adverse Events (Version 4.0; [27]). Elevated lactate dehydrogenase at the initial diagnosis was the strongest predictor for the time interval to systemic treatment initiation with either hydroxyurea or azacitidine, followed by increased bone marrow blast percentage and thrombocytopenia. Elevated lactate dehydrogenase and an increased bone marrow blast percentage are clinical parameters that may indicate pending disease progression in CMML.

Interestingly, neither the hemoglobin level nor red blood cell transfusion-dependence was significantly associated with TTFT in multivariate analysis. This is probably because the initial management of symptomatic anaemia with erythropoiesis-stimulating agents might defer the initiation of treatment with azacitidine or hydroxyurea. In the training set, 26% of CMML patients initially received erythropoiesis-stimulating agents, whereas the highest frequency (44%) was documented in the low-risk group according to our TTFT prediction model. The use of thrombopoietin receptor agonists has not been approved for CMML patients with severe thrombocytopenia so far and as a consequence treatment with either azacitidine or hydroxyurea might be initiated earlier in comparison to patients who present with symptomatic anaemia, who also have erythropoiesis-stimulating agents as a therapeutic option.

In our institution, we generally use a watch and wait strategy in patients with CMML without red blood cell or platelet transfusion dependence and with a hemoglobin level ≥10.0 g/dl and a white blood cell count <20 G/l. Erythropoiesis-stimulating agents are used in patients with CMML-0 or CMML-1 without red blood cell or platelet transfusion dependence and with a hemoglobin level <10.0 g/dl. Hydroxyurea is generally initiated in CMML-0 and CMML-1 patients with a white blood cell count ≥20 G/l with leukocyte dynamics (i. e. rapid increase in in white blood cell count). We generally initiate azacitidine as front-line therapy in patients with CMML-0 or CMML-1 red blood cell and/or platelet transfusion dependence, and/or PLT counts <50 G/l with platelet dynamics (i. e. rapidly dropping platelet counts), if they do not remain stable over a course of several months.

Our proposed TTFT risk model might identify CMML patients who are likely to require early treatment initiation and may be considered for early interventional trials. Another clinical implication of the proposed TTFT risk score is the ability to identify patients who will not require treatment initiation for a longer period of time or who will never require treatment initiation with hypomethylating agents or hydroxyurea and this in turn might help to individualize routine follow-up intervals. We acknowledge the fact that some OS risk scores incorporate molecular information such as ASXL1, NRAS, RUNX1 and SETBP1 mutations in order to estimate OS in CMML [5, 13, 14]. We could not include data of molecular aberrations in our analysis as data collection started in 2004 and molecular studies were not routinely performed at this time. We aimed at creating a simplified score using easily available clinical parameters, which specifically estimates TTFT in CMML. In the great majority (91%) of patients in the validation set, treatment with either azacitidine or hydroxyurea has been initiated, while this was only the case in 62% in the training set. This fact is due to the inclusion criterion of azacitidine treatment for patients included in the Austrian Registry on Hypomethylating Agents, from which most patients (*n* = 60) in the validation set were recruited. Due to this selection bias we could observe more treatment events in the low-risk group of the validation set in comparison to the training set (Fig. 1a,b); however, Kaplan-Meier curves for the intermediate-risk and high-risk groups showed striking similarity between the training set and the validation set.

In summary, we were able to demonstrate that lactate dehydrogenase, bone marrow blast percentage and platelets at the initial diagnosis are the most relevant parameters for the time to first treatment initiation with either azacitidine or hydroxyurea in our CMML cohort. Based on these three parameters, we propose a TTFT risk score for treatment-naïve CMML patients with clinical implications, such as identifying CMML patients for early investigational trials or to tailor individual follow-up intervals. The validity of our model has been confirmed in an external separate CMML set of 65 patients.
